# LBTS‐Net: A fast and accurate CNN model for brain tumour segmentation

**DOI:** 10.1049/htl2.12005

**Published:** 2021-03-16

**Authors:** Mohammed A. M. Abdullah, Sinan Alkassar, Bilal Jebur, Jonathon Chambers

**Affiliations:** ^1^ Computer and Information Engineering Department Ninevah University Mosul Iraq; ^2^ School of Engineering University of Leicester Leicester UK

## Abstract

An accurate tumour segmentation in brain images is a complicated task due to the complext structure and irregular shape of the tumour. In this letter, our contribution is twofold: (1) a lightweight brain tumour segmentation network (LBTS‐Net) is proposed for a fast yet accurate brain tumour segmentation; (2) transfer learning is integrated within the LBTS‐Net to fine‐tune the network and achieve a robust tumour segmentation. To the best of knowledge, this work is amongst the first in the literature which proposes a lightweight and tailored convolution neural network for brain tumour segmentation. The proposed model is based on the VGG architecture in which the number of convolution filters is cut to half in the first layer and the depth‐wise convolution is employed to lighten the VGG‐16 and VGG‐19 networks. Also, the original pixel‐labels in the LBTS‐Net are replaced by the new tumour labels in order to form the classification layer. Experimental results on the BRATS2015 database and comparisons with the state‐of‐the‐art methods confirmed the robustness of the proposed method achieving a global accuracy and a Dice score of 98.11% and 91%, respectively, while being much more computationally efficient due to containing almost half the number of parameters as in the standard VGG network.

## 1 INTRODUCTION

A brain tumour is a mass or growth of abnormal cells which hinders the function of the brain. Brain tumours can vary from low grade glioma (LGG) to high grade glioma (HGG). HGG is more aggressive and has a poorer prognosis compared to LGG [1]. Brain tumours are known to be damaging to surrounding brain tissues, hence it is imperative to assess tumour progress accurately to extend the survival duration of the patients [2]. Magnetic resonance imaging (MRI) is considered as one of the most common modalities for diagnosing brain tumours where various MRI modalities are utilized for the sake of identifying brain tissue including T1, T2 and fluid attenuation inversion recovery (FLAIR). Each of the aforementioned MRI modalities have different relaxation time and hence are used to identify different brain tissues [3].

To this end, isolating the tumour from the healthy tissue is termed as tumour segmentation [4]. In essence, tumour segmentation helps in tracking the progress of the tumour and assess whether the tumour enlarges or shrinks in response to the treatment. Although surgery is the most common treatment for brain tumours, radiation and chemotherapy are often employed as a line of treatment when the tumour is not accessible or its removal may damage surrounding healthy tissues. They are also employed after surgery to reduce the chances of tumour re‐growth. Tumour segmentation is a challenging task due to complexity and variation of the tumour structure as well as the intensity similarity between tumour tissues and normal brain tissues. While some tumours such as meningiomas can be easily isolated, others like gliomas and glioblastomas are troublesomes [2]. This makes manual tumour segmentation a tedious task and in some occasions variations can be seen in the segmentation results of oncologists owing to the heterogeneous appearance and fuzzy shape of the tumour. Therefore, it is imperative to develop automatic segmentation algorithms to ease this challenging task.

Generally speaking, segmentation models can be universally classified as either generative models or discriminative models. While generative models segment objects by relying on the appearance, illumination and occlusion of the shape; they are difficult to design and their accuracy is generally low, especially for complex objects with inhomogeneous textures [5] such as brain tumours. On the other hand, discriminative models are easier to be generalized as they depend on the low‐level image features to be learned from the annotated training images in order to accurately capture local appearance variations. In principle, deep neural networks (DNNs) belong to the discriminative models as feature learning occur without prior information about the segmented object. DNN relies on image intensities which are fed to the multi‐convolution layers to generate the desired output without depending on handcrafted feature. Therefore, a DNN can be generalized to different types of image with an outstanding accuracy in terms of a segmentation/recognition task. In this context, different CNN models have been proposed in the literature for medical image segmentation such as SegNet, GoogLeNet and AlexNet [6]. It is worth pointing out that the great accuracy, attained by CNNs, comes with an increased computational complexity and storage consumption. Besides, complex CNN architectures require a powerful GPU, which consumes high power and may generate too much heat. Moreover, these complex CNN architectures are difficult to implement in a low‐end PC or embedded architectures including smartphones. Hence, it is necessary to lighten deep learning networks in order to extend CNNs to low‐cost architectures and increase the applicability of these methods.

Our contributions in this letter can be summarized as follows. (1) a lightweight brain tumour segmentation network (LBTS‐Net) is proposed for a robust yet fast tumour segmentation; (2) transfer learning is integrated within the LBTS‐Net to fine‐tune the base VGG network to achieve a robust tumour segmentation. To the best of our knowledge, this work is amongst the first in the literature which the VGG‐16 and VGG‐19 networks are lightened and tailored for brain tumour segmentation. This letter is organized as follows. Section 2 presents the related work in terms of CNNs and brain tumour segmentation. Section 3 describes the proposed method for brain tumour segmentation. This is followed by the experimental results and comparisons with the state‐of‐the‐art work in Section 4. Finally, Section 5 concludes this letter.

## 2 RELATED WORK

There has been an increasing amount of work related to brain tumour segmentation in the last years. This is imposed by the importance of this topic across the medical society [2]. Various researchers have adopted a DNN as the core of their brain tumour segmentation method. As a case in point, the authors in [3] proposed a method for brain tumour segmentation using a CNN where global and local features are fed to the network using two‐phase training procedures. Similarly, the work in [7] proposed a method for HGG and LGG tumour segmentation using a patch‐based CNN with a reduced filter kernel. In [8] the authors integrated the conditional random fields (CRF) with a fully convolutional network to establish an accurate tumour segmentation. A 3D CNN architecture with 11 learnable layers is utilized in [9] to achieve efficient tumour segmentation. In our previous work [10], we have exploited the VGG16 network along with transfer learning for brain tumour segmentation.

Despite that the aforementioned methods improved the segmentation results; however, this improvement comes at the expense of increasing the computational complexity and storage requirement. Therefore, decreasing the complexity of CNN for tumour segmentation is essential and further work is required to develop an accurate and fast brain segmentation method.

As far as we know, lightening a CNN has not been well studied for brain tumour segmentation. There are some attempts in the literature to lighten CNN for image recognition or semantic segmentation tasks. For example, Zhao et al. [11] proposed a lightweight CNN for finger vein recognition using fewer convolution layers to gather the finger features. The authors reported reduction in the training time and resource usage using this method. Lai et al. [12] proposed a lightweight CNN approach for semantic image segmentation. They proposed to reduce the convolution filter size and decrease the number of input channels to make the model lighter. Similarly, the authors in [13] proposed a lightweight CNN for real‐time semantic segmentation. The proposed model has multiple branches of skipped connections to reduce the network parameters and increase the speed. Ding et al. [14] proposed a method to lighten the Unet network for brain tumour segmentation. The simple reducing‐net is proposed which is the basic block of their method where only one convolution operation is performed before each down‐sampling process. The authors reported a reduction in the parameters by a factor of 4/5 compared to the standard Unet. However, the reported accuracy of their work is relatively low compared to our work. In this letter, an accurate yet lightweight brain tumour segmentation method is proposed and is explained in the next section.

## 3 PROPOSED METHOD

The proposed method for tumour segmentation includes two parts: (1) a lightweight CNN for tumour brain segmentation which is specially designed to decrease the number of parameters and save storage requirements. (2) a transfer learning strategy including pre‐training and fine‐tuning to tailor the network and achieve an accurate brain tumour segmentation. The details of these two parts are given in the following sub‐sections.

### 3.1 LBTS‐Net

Various CNNs architecture are available off‐the‐shelf such as Alex‐Net, Google‐Net, Inception‐Net and VGG‐Net. However, several works confirmed that the VGG models achieve a promising performance when dealing with medical imaging [15, 16, 17] as opposed to other CNN architecture. Therefore, we have chosen the VGG models to be the backbone network of our proposed model. Despite the great accuracy offered by the VGG models, they are very computationally demanding in terms of the number of parameters and storage resources. Therefore, we propose a lightweight VGG models to address the aforementioned problem.

**FIGURE 1:**
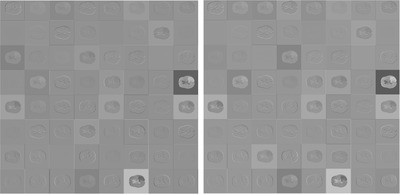
The activations of the first convolution layer in the VGG‐16 network (left) and VGG‐19 network (right)

In this letter, two models have been proposed, namely: LBTS‐Net‐16 and LBTS‐Net19, through lighting the VGG‐16 and VGG‐19 networks by reducing the number of convolution filters and adapting depth‐wise separable convolution. In this context, the proposed LBTS‐Net contains 32 convolution filters, which is half the number of filters in the original VGG‐16 or VGG‐19 networks. This is because the activations in this layer have redundant information with similar structures and patterns for brain images as can be seen in Figure 1. From this view, it can be inferred that reducing the number of convolution filters in this layer will not hinder the segmentation accuracy significantly as will be shown in the experiments section. The structures of the LBTS‐Net16 and LBTS‐Net19 are illustrated in Table 1. In addition, to reduce the number of parameters in the LBTS‐Net, depth‐wise separable convolution is exploited instead of the standard convolution in the first layers (layers 2–4) of this network. The depth‐wise separable convolution is a lightweight version of full convolutions. Basically, the initial convolution layers usually contain low‐level information represented by edges and their combinations which are considered as low‐level functions. These low‐level functions are simpler than the following layers therefore replacing the full convolution operations in these layers with the depth‐wise convolutions will not deteriorate their designated function.

**TABLE 1:** Comparison of the VGG‐16 and VGG‐19 with the LBTS‐Net16 and LBTS‐Net19 architectures in terms of accuracy and complexity where “DW‐Conv” represents the depth‐wise convolution operation

Layer	VGG‐19	LBTS‐Net19	VGG‐16	LBTS‐Net16
Layer 1	Conv,64	Conv,32	Conv,64	Conv,32
Layer 2	Conv,64	DW Conv,32	Conv,64	DW Conv,32
Layer 3	Conv,128	DW Conv,64	Conv,128	DW Conv,64
Layer 4	Conv,128	DW Conv,64	Conv,128	DW Conv,64
Layer 5	Conv,256	Conv,256	Conv,256	Conv,256
Layer 6	Conv,256	Conv,256	Conv,256	Conv,256
Layer 7	Conv,256	Conv,256	Conv,256	Conv,256
Layer 8	Conv,256	Conv,256	Conv,512	Conv,512
Layer 9	Conv,512	Conv,512	Conv,512	Conv,512
Layer 10	Conv,512	Conv,512	Conv,512	Conv,512
Layer 11	Conv,512	Conv,512	Conv,512	Conv,512
Layer 12	Conv,512	Conv,512	Conv,512	Conv,512
Layer 13	Conv,512	Conv,512	Conv,512	Conv,512
Layer 14	Conv,512	Conv,512	Fc,4096	Fc,4096
Layer 15	Conv,512	Conv,512	Fc,4096	Fc,4096
Layer 16	Conv,512	Conv,512	output layer with softmax	output layer with softmax
Layer 17	Fc,4096	Fc,4096	–	–
Layer 18	Fc,4096	Fc,4096	–	–
Layer 19	output layer with softmax	output layer with softmax	–	–
**# of parameters**	1.43 × 10^8^	7 × 10^7^	1.38 × 10^8^	6.5 × 10^7^
**Memory**	**549 Mb**	**269 Mb**	**528 Mb**	**248 Mb**
**Accuracy**	**98.66%**	**98.32%**	**98.46%**	**98.11%**

### 3.2 Transfer learning and implementation details

The second part of the proposed method includes transfer learning. Transfer learning has recently been reported to achieve an outstanding image segmentation performance [18]. In this work, an accurate tumour segmentation is achieved via fine‐tuning the proposed LBTS‐Net using the BRATA2015 dataset. Tumour images and their corresponding pixel labels are fed the DNN to train this network to recognize the new labels and establish the semantic segmentation. Next, the label layer of the VGG SegNet has been replaced with the pre‐defined classes and their corresponding class weights to establish the soft‐max classification layer.

**FIGURE 2:**
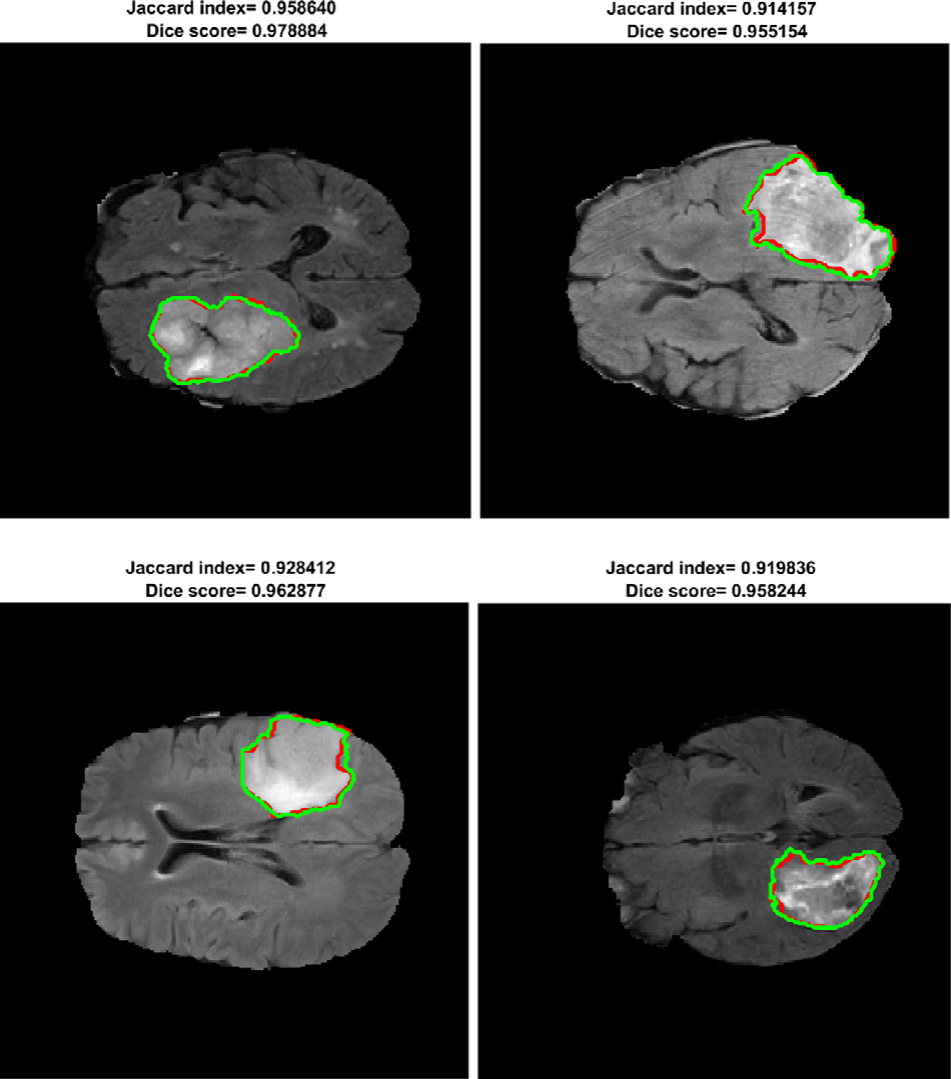
Results of brain tumour segmentation using the proposed method where the green line denotes the ground truth while red line is the segmentation boundary of the proposed method

The architecture of the proposed network is shown in Table 1. The LBTS‐Net16 has 13 convolutional layers while the LBST‐net19 has 16. Each layer has a filter bank, element‐wise tanh non‐linearity, max‐pooling and sub‐sampling to build up the feature map sets. To start with, a convolution with a filter bank is performed to generate the feature map which is then batched‐normalized. The (ReLU) activation function is used in this work which can be defined as max(0,x). This is followed by sub‐sampling the output by a factor of 2 using a max pooling process with a 2 × 2 non‐overlapping window. As a result of the many sub‐sampling processes and max‐pooling layers in the encoder network, spatial resolution of the feature map will be affected. This will result in poor boundary details which will hinder the segmentation process. In order to mitigate this problem, the indices of the max‐pooling for each encoder feature map are stored and these indices are utilized later in the decoder stage to up‐sample the corresponding feature map and hence generate the sparse feature representation. Next, a trainable decoder filter bank is convolved with these feature sets to amplify the sparse feature sets. Later, these feature sets are forwarded to the soft‐max classification layer to classify the pixels in the tumour image into either a tumour or a brain pixel. To improve the network accuracy and avoid under‐fitting, image data augmentation is used. In this regard, different image manipulations have been used such as: rotation, translation and random reflection. Also, for the sake of improving the training process, the class weights have been balanced. Figure 2 depicts segmentation results of the proposed method on images from the BRATS database.

The implementation details of the network are as follow. the initial learning rate and L2 regularization have been empirically set to ($1e^{-3}$, and $5e{}^{-}4$), respectively. A momentum term equal to 0.9 is used as an optimization procedure with the stochastic gradient descent with momentum (sgdm) and the cross‐entropy loss function. The batch‐size is set to 64 and the network converged after 30 epochs.

## 4 EXPERIMENTAL RESULTS

Our experiments are evaluated on the multimodal brain tumour segmentation challenge (BRATS) 2015 database [19]. The database includes synthetic and clinical images of brain tumours. The brain images are acquired in multi‐contrast MRI scans including T1, T1‐weighted contrast‐enhanced, T2‐weighted, and FLAIR contrast. The clinical and synthetic tumour images have both been marked by experts to generate the ground truth data which are later used to measure the accuracy.

### 4.1 Comparisons and discussion

**FIGURE 3:**
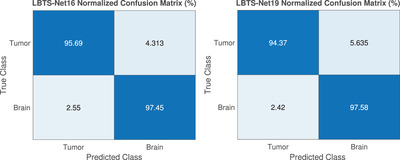
The confusion matrix of the proposed method LBTS‐Net16 (left) and LBTS‐Net19 (right)

All experiments are evaluated on a 2.6 GHz core i7 processor with 16 Gb of RAM and Nvidia GTX 1660 Ti GPU under the MATLAB environment. 70% of the MRI images are used for training while the rest are employed for testing. The results are generated based on the HGG images from the BRATS 2015 database considering the whole tumour region. The whole tumour regions are represented by the concatenation of all tumour labels namely: necrotic, non‐enhancing tumour, peritumoural edema and enhancing tumour. The confusion matrix for both the tumour and brain which provides a visualization of the semantic segmentation performance is generated as shown in Figure 3. Moreover, different measures are utilized to measure the performance of the proposed including global accuracy, mean accuracy and Dice score which are recorded to be 98.11%, 96.86% and 91%, respectively, for the LBTS‐Net16.

In order to illustrate the effect of reducing the number of filters and using the depth‐wise convolution in the first layers, we conducted comparisons with both the standard VGG‐16 and VGG‐19 networks which are trained on the same images under the same conditions. Table 1 illustrates the structure and global accuracy of the proposed models (LBTS‐Net16 and LBTS‐Net19) versus those of VGG‐16 and VGG‐19 networks. Despite the accuracy of the proposed LBTS‐Net16 slightly dropping by less than 0.5% compared to the baseline VGG‐16, a significant saving in the number of parameters and required memory is achieved. Similarly, for the LBTS‐Net19, the accuracy of the proposed model is comparable to the original VGG‐19, while the number of parameters and storage consumption are almost halved. This will significantly decrease the computational and storage resources required during the network training and testing, which in turn will greatly reduce the complexity of the network. It can be concluded from Table 1 that the performance of the LBTS‐Net16 is comparable to that of LBTS‐Net19 while the complexity is lower. Therefore, we adopted LBTS‐Net16 in our experiments. Moreover, in order to evaluate the performance of the proposed method against similar state‐of‐the‐art work on the BRATS database, Table 2 illustrates the mean Dice score and architecture of the aforementioned works. It can be clearly seen from Table 2 that the proposed method outperforms similar works while being much lighter in terms of network parameters and storage space. Owing to the lightweight architecture of our model, the proposed architecture contains almost 50% fewer parameters compared to the standard VGG networks while achieving similar performance. With that said, the proposed network is feasible in low‐performance PCs.

**TABLE 2:** Performance evaluation of the state‐of‐the‐art methods and the proposed method using the BRATS database

Segmentation Method	Dice score (%)	Details
Pereira 2016 [7]	88	Patch‐based segmentation net with a reduced filter kernel
Havaie 2017 [3]	88	A multi‐scale CNN for patch‐based segmentation
Kamnitsas 2017 [20]	90.1	3D multi‐scale CNN for patch‐based segmentation with optimized softmax layer
Chang 2019 [21]	80	CNN combined with a fully connected CRF as a mixture model to introduce the global context information
Ding SRNET 2019 [14]	83	Stack multi‐connection simple reducing‐net (SMCSRNet)
Khan 2020 [22]	81	Handcrafted features including LBP and HOG are combined with CNN to achieve pixel classification
Alkassar et al. 2019 [10]	89	VGG16 is utilized for brain tumour segmentation along with transfer learning
**Proposed (LBTS‐Net16)**	91	Lightweight VGG‐16 with reduced number of convolution filters and depth‐wise convolution
**Proposed (LBTS‐Net19)**	91.5	Lightweight VGG‐19 with reduced number of convolution filters and depth‐wise convolution

## 5 CONCLUSIONS

In this letter, two lightweight CNN models (LBTS‐Net16 and LBTS‐Net19) were proposed for brain tumour segmentation. The proposed models were based on lightening the VGG architecture by reducing the number of redundant convolution filters in the first layer and adopting the depth‐wise convolution in the initial layers of the network (layers 2–4). Transfer learning was also utlilised to fine‐tune the proposed method to achieve a robust segmentation. Experimental results demonstrated that the proposed method significantly reduced the computation complexity of the network while maintaining a similar performance compared to the base‐line VGG architecture. In addition, experimental results showed that the proposed model achieved promising performance which outperformed similar methods.

## CONFLICT OF INTEREST

The authors declare no conflict of interest.
